# Are the Hands of Veterinary Staff a Reservoir for Antimicrobial-Resistant Bacteria? A Randomized Study to Evaluate Two Hand Hygiene Rubs in a Veterinary Hospital

**DOI:** 10.1089/mdr.2018.0183

**Published:** 2018-12-08

**Authors:** Eva Espadale, Gina Pinchbeck, Nicola J. Williams, Dorina Timofte, K. Marie McIntyre, Vanessa M. Schmidt

**Affiliations:** ^1^ Department of Small Animal Clinical Science, Institute of Veterinary Science, University of Liverpool, Neston, United Kingdom.; ^2^ Department of Epidemiology and Population Health, Institute of Infection and Global Health, University of Liverpool, Neston, United Kingdom.; ^3^ Department of Infection Biology, Institute of Infection and Global Health, University of Liverpool, Neston, United Kingdom.

**Keywords:** hand hygiene, antimicrobial-resistant bacteria, alcohol hand hygiene rub, lactic acid hand hygiene rub

## Abstract

Hand hygiene (HH) is the most successful intervention for hospital infection control. HH rubs with residual action are desired. This study aimed to compare the efficacy of alcohol (A-HH) and lactic acid (LA-HH) rubs, with the latter being marketed as having residual activity. We investigated reductions in bacterial colony-forming units (CFUs), prevalence of antimicrobial-resistant (AMR) organisms, and risk factors for increased counts on the hands of veterinary staff. A randomized, crossover study (53 individuals) was performed in a referral veterinary teaching hospital. Hand plates were taken before, immediately after, and 6 hours after HH. A blinded investigator counted CFUs per plate. Methicillin-resistant *Staphylococcus aureus*/*pseudintermedius* (MRSA/MRSP), Enterobacteriaceae, and *Pseudomonas* species (spp.) were characterized. Gender, profession, time point, and HH product were included as variables within multivariable analyses. A significant reduction in bacterial CFU was seen immediately after A-HH rub application (*p* < 0.001); however, neither product showed any significant residual action. Veterinarians had higher bacterial CFUs than nurses (*p* = 0.005); contact with patients, rather than the environment, was also associated with higher counts (*p* < 0.001). MRSA, MRSP, Enterobacteriaceae spp., and *Pseudomonas* spp. were detected on 7%, 2%, 14%, and 2% of study participant's hands (*n* = 208 samples), respectively. Frequent HH administration using an A-HH rub was effective at reducing bacterial CFU on hands *in vivo* in this veterinary hospital setting, but its use needs further encouragement in veterinary staff. The high prevalence of antimicrobial bacteria on hands is of concern; they might act as reservoirs for patients, the environment, and in-contact people.

## Introduction

Commensal bacteria of the skin, mucosa, or gastrointestinal tract, including staphylococci and Enterobacteriaceae, are often opportunistic pathogens.^1–3^ Moreover, antimicrobial resistance (AMR) is increasing among such pathogens.^4,5^ In particular, methicillin-resistant coagulase-positive staphylococci (MR-CoPS) such as *S. aureus* (MRSA) and *S. pseudintermedius* (MRSP) and extended-spectrum beta-lactamase- and AmpC-producing-Enterobacteriaceae have emerged and are disseminated within veterinary hospitals.^6,7^ Carriage of the *mecA* gene confers resistance to all beta-lactam antibiotics in staphylococci.^8^ In Enterobacteriaceae*,* genes conferring resistance to third-generation cephalosporins (*e.g*., *bla*_CTX-M, TEM, SHV, OXA,_ and AmpC-type) may reside on large conjugative plasmids and are hence able to transfer readily between bacteria by horizontal transmission.^9^

Dissemination of AMR bacteria, particularly those with multidrug resistance (MDR—resistance to at least one agent from three or more antimicrobial classes),^10^ within the veterinary hospital environment, can lead to serious nosocomial infections in already immune-compromised animals. This may lead to increased morbidity, mortality, and associated costs, and may also represent a public health risk to staff and owners.^11^ Although the true prevalence of nosocomial infections in veterinary hospitals has not been well established,^12,13^ a considerable number of outbreaks caused by AMR bacteria have been documented.^14–16^ One of the largest involving 63 confirmed cases of MRSP occurred in a Finnish veterinary teaching hospital between 2010 and 2012.^17^ Therapeutic options for treating infections with MDR bacteria in veterinary patients are limited and nonlicensed, expensive drugs with potential side effects may need to be used. Such drugs may lack pharmacokinetic data,^18^ and/or be considered “critically important” for human medicine by the World Health Organisation.^19^ Due to these limitations and because the hands of clinical staff are the most common vehicle for transmission of microorganisms from patient to patient within the healthcare environment,^20^ hospital biosecurity programs that reduce overall burden and transfer of microbes are of paramount importance.

Hand hygiene (HH) is reportedly the most successful intervention for hospital infection control in humans.^21^ Hand rubbing with an alcohol solution has been shown to be more effective, for decontamination of healthcare workers' hands, than handwashing with an unmedicated soap.^22^ Alcohol-based HH rubs rapidly kill microorganisms by denaturing and coagulating proteins, and disrupting the cellular membrane, leading to lysis.^23^ While being convenient, their reported disadvantages include inactivity in the presence of organic matter, potential skin irritation and discomfort when used on broken skin, their flammable nature, toxicity if ingested, enhanced staphylococcal biofilm production *in vitro*,^24^ and no residual action (*i.e*., further impact on microorganisms after HH is administered).^25^ Furthermore, alcohol hand rubs have no impact on bacterial spores or protozoan oocysts, and have poor impacts on some nonenveloped viruses.^21^

Veterinary staff compliance with hospital HH protocols is often poor.^26^ For example, only 76 of 182 (41.7%) veterinary technicians and support staff in a small animal private practice reported washing their hands between patients, purportedly due to time constraints.^27^ Therefore, a product with residual antimicrobial activity may offer additional benefits within healthcare environments. Lactic acid is a promising biodegradable and nontoxic antimicrobial product suitable for healthcare applications, and is marketed for this purpose. It is lytic to bacterial membranes and shows broad-spectrum antimicrobial activity impacting AMR and MDR bacteria *in vitro.*^28^ To the authors' best knowledge, however, its *in vivo* efficacy as an HH rub has not been evaluated in either human or veterinary hospitals. Moreover, there is limited published research regarding the use of HH in veterinary facilities.^11,25,27^ Studies comparing the microbiological efficacy of different hand rub products on hand contamination of veterinary personnel are sparse and limited to testing single applications of hand rub products.^29^

The aims of this study were, therefore, as follows: (1) to investigate the efficacy of hand hygiene (HH) rubs in the veterinary clinical setting by comparing a rub containing lactic acid (recently marketed as having residual antimicrobial activity and hypothesized to be at least as efficacious as ethanol) against ethanol, in terms of reduction in bacterial counts; (2) to examine risk factors of bacterial hand contamination of veterinary staff; and (3) to investigate the prevalence of AMR potential pathogenic bacterial contamination on the hands of study participants.

## Methods

### Setting and study participants

A randomized controlled crossover study design was used over a 2-week period. The primary outcome measure was colony-forming units (CFU) on hands of participants after performing HH and 6–8 hours later, just before they left the work place. Secondary outcomes include the presence or absence of AMR among potential pathogenic bacteria contaminating individuals' hands.

Power analysis was used to ascertain the number of study participants needed using a pilot study with 10 volunteers (Supplemental Data; Supplementary Data are available online at www.liebertpub.com/mdr). To detect a difference of 60 CFU or greater per participant between experimental groups after using the HH rubs, with a power of 0.8 and a standard deviation of 106.8, a sample size of 51 people was estimated as necessary. Fifty-three volunteers were recruited from a small animal university teaching hospital. Volunteers were allocated to groups, stratified by profession. Only staff and students scheduled to be at the teaching hospital for the 2-week study duration were included. Covariates collected included profession (veterinarian, nurse or auxiliary, administrative staff, and student), gender, and activity before performing HH (*e.g*., animal contact or environmental contact—using a computer or phone). In addition, veterinarians were further classified as surgeons or nonsurgeons. All enrolled volunteers provided informed written consent before participation. The study was approved by the University of Liverpool Veterinary Ethics Committee (reference VREC439).

### Study protocol and HH procedures

Volunteers were randomly allocated (using a computer-generated code) to use one of two HH products during week 1. The following week, they used the alternative HH product. The participants were aware of which product they were using each week (*i.e*., they were not blinded). All volunteers were briefed on the study and educated regarding HH techniques and finger-plate sampling methods before the study was implemented. The participants used the designated HH rubs throughout the day. They were asked to apply 2–3 mL (two pumps) of the product on their hands (according to the manufacturer's instructions) and rub both hands together on all surfaces until dry, without using paper towels. The participants washed their hands with soap and water when they were visibly soiled, after which the HH rub was immediately reapplied. The whole procedure was performed according to WHO HH technique recommendations.^21^

The lactic acid-based (LA-HH) rub (Viridis^®^) contained lactic acid 0.3%, inert material, surfactant, and water; the alcohol-based (A-HH) rub contained 70% ethanol, carbomer, isopropyl myristate, glycerin, monopropylene glycol, vitamin E, and demineralized water. At the start of each day, participants were each given three code-labeled Columbia 5% defibrinated horse blood agar (CAB) plates supplemented with neutralizer (30 mL/L Tween 80, 30 g/L Saponin, 1 g/L L-histidine, and 1 g/L L-cysteine) as previously described.^32^ They were asked to provide imprints of their dominant hand (finger tips and thumb) on three occasions: immediately after performing a clinical/nonclinical procedure in the morning (T1), immediately after using the HH rub following that procedure (T2), and 6–8 hours later, just before leaving the work place (T3). This was repeated on 3 consecutive days in each test week, providing nine plates per participant for each product over the course of the study.

At the end of each day, the CAB plates obtained at T1, T2, and T3 were checked for growth before being incubated aerobically overnight at 37°C. The number of CFUs per plate was counted the following morning. The maximum count was 300 CFUs/plate. If counts were above this, confluence was considered and a bacterial count of 350 CFUs was assigned to the sample.^22^ The researcher who read the plates was blinded to the HH product used.

### Characterization of isolates

All colonies obtained from the three plates on 3 consecutive days from each participant and product at T1 and T3 were pooled for further analysis. Colonies were transferred from CAB into tryptone soy broth for enrichment to increase the sensitivity of detection and were then incubated aerobically overnight at 37°C. Overnight broth cultures were inoculated onto selective agar primed to identify third-generation cephalosporin-resistant (3GCR) Enterobacteriaceae (Eosin Methylene Blue with cefotaxime (1 mg/L); EMBAcx), MR-CoPS (Chromogenic Brilliance Agar; CBA), and *Pseudomonas* spp. (*Pseudomonas* CN 200 mg/L [Cetrimide and 15 mg/L Sodium Nalidixate]) and incubated aerobically overnight at 37°C. Three random colonies with typical morphology for Enterobacteriaceae on EMBAcx or *Pseudomonas* spp. on *Pseudomonas* CN were selected from each agar plate if available and transferred onto nutrient agar (NA); three random colonies with typical morphology for hemolytic *Staphylococcus* spp. were selected from CBA plates if available and transferred onto CAB. All plates were incubated aerobically overnight at 37°C and the isolates obtained were included for further analysis in this study. Gram staining, biochemical tests (catalase and oxidase for both staphylococcal and Enterobacteriaceae isolates, and free [rabbit plasma] and bound [Prolex™; Prolab, Wirral, United Kingdom] coagulase for *Staphylococcus* spp.), as well as polymerase chain reaction (PCR) assays (*nuc* gene for coagulase-positive *Staphylococcus* spp. and *uid*A and *usp*A genes for Enterobacteriaceae)^30,31^ were undertaken to investigate bacterial genus and species. All media were sourced from LabM Ltd (Bury, United Kingdom) and Oxoid Ltd (Basingstoke, United Kingdom).

### Antimicrobial susceptibility testing

All isolates were subjected to disc diffusion testing to determine antimicrobial susceptibility in accordance with the Clinical and Laboratory Standards Institute (CLSI).^32^ For *Staphylococcus* spp. the following discs were used: 1 μg oxacillin (OX), 30 μg cefoxitin (FOX), 2 μg clindamycin (CD), 15 μg erythromycin (E), 1.25 μg/23.7 μg trimethoprim-sulfamethoxazole (TS/STX), 30 μg chloramphenicol (C), 30 μg tetracycline (T), 10 μg fusidic acid (FA), 10 μg gentamicin (GM), 5 μg rifampin (RP), 5 μg enrofloxacin (ENR), 5 μg marbofloxacin (MAR), and 30 μg amikacin (AK). For Enterobacteriaceae, the following discs were used: 10 μg ampicillin (AP), 20 μg/10 μg amoxicillin clavulanate (AUG), 10 μg gentamicin (GM), 30 μg chloramphenicol (C), 1.25 μg/23.7 μg trimethoprim-sulfamethoxazole (STX), 30 μg tetracycline (T), 5 μg enrofloxacin (ENR), and 5 μg marbofloxacin (MAR); Enterobacteriaceae were also tested against cefpodoxime (CPD; 10 μg) as an indicator of 3GCR resistance. Published breakpoints were used to classify isolates as either resistant or not resistant according to CLSI 2015 guidelines for animal pathogens.^32^ Where these breakpoints were not available, human breakpoints (European Committee on Antimicrobial Susceptibility Testing, EUCAST) were used instead.^33^ Isolates resistant to at least one tested antimicrobial were defined as antimicrobial resistant (AMR) and isolates resistant to at least one agent from three or more antimicrobial classes were defined as MDR.^10^ All the discs were purchased from Oxoid Ltd. (Basingstoke, United Kingdom), except for MAR, which was obtained from Vetoquinol Ltd. (Towcester, United Kingdom).

### Characterization of antimicrobial resistance genes

The presence of resistance genes was investigated for all isolates using PCR assays to identify the following: *mecA* for *Staphylococcus* spp.^34^, *bla*_CTX-M,_^35^ *bla*_SHV_, *bla*_TEM_ and *bla*_OXA,_^36^ *bla*_AmpC_^37^ for Eutero-bacteriaceae, for and for co-carriage of plasmid-mediated fluoroquinolone resistance (*qnr*A, *qnr*B, and *qnr*S)^38^ in 3GCR isolates. All PCR assays were performed and analyzed as previously reported.^39,40^ Positive control strains were included and molecular grade water (Sigma-Aldrich Company Ltd., Gillingham, United Kingdom) was used as the negative control. All primers were synthesized by Eurofins MGW Operon (Ebersberg, Germany).

**FIG. 1.**
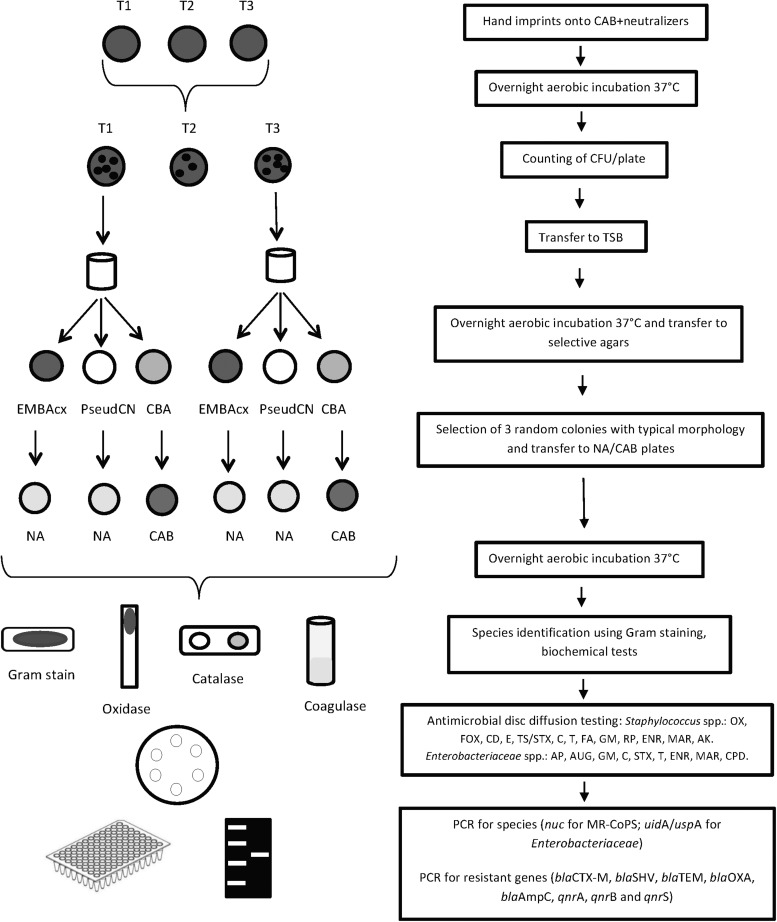
Schematic view of methods. CAB, Columbia 5% defibrinated horse blood agar; CFU, colony-forming units; TSB, tryptone soy broth; EMBAcx, Eosin Methylene Blue with cefotaxime (1 mg/L); PseudCN, *Pseudomonas* CN 200 mg/L (Cetrimide and 15 mg/L Sodium Nalidixate); CBA, Chromogenic Brilliance Agar; NA, nutrient agar; OX, oxacillin; FOX, cefoxitin; CD, clindamycin; E, erythromycin; TS/STX, trimethoprim-sulfamethoxazole; C, chloramphenicol; T, tetracycline; FA, fusidic acid; GM, gentamicin; RP, rifampin; ENR, enrofloxacin; MAR, marbofloxacin; AK, amikacin; AP, ampicillin; AUG, amoxicillin clavulanate; CPD, cefpodoxime.

A schematic view of the methods is represented in Figure 1.

### Statistical analysis

CFU data were not normally distributed, hence either log_10_ transformed or nonparametric test data were used for analyses.

CFU obtained on 3 consecutive days each week were averaged per participant and product at each time point (T1, T2, and T3). A paired nonparametric Wilcoxon signed rank test was used to compare participants' median CFU before and after using the two HH products. McNemar's test was used to detect possible differences between the prevalence of AMR bacteria on the hands of study participants at different time points. Data were analyzed using the SPSS^®^ statistical package (SPSS 24.0 for Windows^®^; SPSS, Inc., Chicago, IL). A *p* value <0.05 was considered significant.

Multilevel multivariable linear regression modeling was used to examine the effect of HH product, time point, and other covariates on log_10_ CFU, adjusting for clustering of samples within participants by their inclusion as a random intercept. Univariable analyses followed by manual backward-stepwise procedures were used on a full model and variables with a *p*-value <0.05 were retained in the model. The significance of appropriate interaction terms was examined for all fixed-effect variables. Model residuals were evaluated for normality. Data were analyzed using the MLwiN statistical software package (MLwiN Version 2.1 Centre for Multilevel Modelling, University of Bristol, United Kingdom).

Data for AMR bacterial prevalence (resistance to at least one tested antimicrobial) were collapsed to participant level such that a participant with at least one resistant isolate (at each time point) was classed as resistant within analyses.

## Results

### Comparison of two HH products in the veterinary hospital

#### Participants' characteristics

Fifty-three participants (33 females and 20 males) were involved in the study and provided data for at least one time point: 4 reception and administrative staff, 4 animal care auxiliaries, 12 nurses, 14 veterinary students, and 19 veterinarians. Five of the veterinarians were surgeons.

One participant provided only the 9 samples for the A-HH rub due to illness during the second week of the study (participant 12, see overview Supplementary Table S1). Another participant (participant 8, see overview Supplementary Table S1) left the study after 1 day (this participant provided three samples for the LA-HH rub) due to severe skin irritation after LA-HH application. Four other participants failed to provide the samples on one of the study days due to single-day work absences. Finally, six samples were missing from different participants at various time points. Five of these samples were from T3 (just before going home) due to the participants forgetting to perform the imprint at the end of the day; the remaining missing sample was from T2 (the participant lost the sample). At the end of the 2 weeks, 452 samples from 52 participants were obtained from the LA-HH group and 460 samples from 52 participants were obtained from the A-HH rub (total of 912 samples). The total number of participants providing paired data for statistical analysis comparing HH rubs was 51.

#### Hand contamination before and after use of HH products

Overall, 51 participants provided samples for both tested products (paired data). Table 1 illustrates median CFU on participants' hands at different time points. There was a significant reduction in CFU immediately after using A-HH (*p* < 0.001), and a significant increase in CFU immediately after using LA-HH. At the end of the day (T3), median CFU was not significantly different from T1 for either product.

**Table 1.** Microbiological Efficacy of Two Hand Hygiene Products on Colony-Forming Units in 51 Study Participants Who Provided Paired Data for Analysis

	*Median CFU per hand*^a^*(interquartile range)*					
	*T1*	*T2*	*T3*	p *value*^c^	p *value*^d^	p *value*^e^
Lactic acid *n* = 51	84 (156)	209 (178)	83 (133)	**<0.001**	**<0.001**	0.222
Alcohol *n* = 51	88 (113)	19 (52)	85 (138)	**<0.001**	**<0.001**	0.417
*p* value^b^	0.781	**<0.001**	0.647			

Bold values indicate *p* < 0.05.

^a^ Dominant hand; *n* = total number of participants providing paired data for statistical analysis comparing the HH rubs; T1—after patient/environmental contact and before HH, T2—immediately after HH, T3—6–8 hours later, just before going home.

^b^ *p* value for Wilcoxon signed rank test (Wilcox) comparing CFU for lactic acid and alcohol rub groups at T1, T2, and T3.

^c^ *p* value for Wilcox comparing CFUs for products separately between T1 and T2.

^d^ *p* value for Wilcox comparing CFUs for products separately between T2 and T3.

^e^ *p*-Value for Wilcox comparing CFUs for products separately between T1 and T3.

CFU, colony-forming units.

#### Risk factors for hand contamination

Multilevel univariable analyses from 53 participants suggested there was no significant association between (log) CFU counts and either gender (*p* = 0.67) or being a surgeon (*p* = 0.56). Furthermore, there was no significant difference between the effects of the two HH products and the week day on which they were used (*p* = 0.9) or which product was used first (*p* = 0.61) (Supplementary Table S3).

Multilevel multivariable analysis (Table 2) showed that there was a significant effect of product interacting with time point on (log) CFU; the alcohol HH product lowered (log) CFU at T2 only (*p* < 0.001). In addition (log), CFU was significantly higher after contact with animals compared with environmental contact (*p* = 0.002), and being a veterinarian was associated with higher bacterial counts on hands compared to nurses (*p* = 0.005).

**Table 2.** Multilevel, Multivariable Regression Model Describing Factors Associated with Overall Hand Contamination After Hands Had Been Treated Using Either Lactic Acid- or Alcohol-Based Hand Hygiene Products

*Variables*	*Beta*	*SE*	*Wald* p *value*
Intercept	1.41	0.11	—
Product			0.9
Alcohol	Ref	—	—
Lactic acid	−0.001	0.06	
Type of contact			**<0.001**
Environment	Ref	—	—
Animal	0.214	0.05	
Job			**0.002**
Nurse	Ref	—	—
Administration staff	0.388	0.201	
Auxiliaries	0.394	0.209	
Veterinarians	0.358	0.127	
Students	0.113	0.143	
Time			**<0.001**
T1	Ref	—	—
T2	−0.75	0.06	
T3	−0.18	0.06	
Time^*^product interaction			**<0.001**
Lactic acid^*^ T1	Ref	—	—
Lactic acid^*^ T2	1.04	0.09	
Lactic acid^*^T3	0.08	0.09	

The outcome is the log colony-forming units, and the model includes clustering within participants (53 participants and 912 samples).

Ref, used as the baseline reference in the multilevel modeling; SE, standard error; bold values indicate *p* < 0.05. T1—after patient/environmental contact and before HH, T2—immediately after HH, T3—6–8 hours later, just before going home. The log CFU at each time point for each product after taking into account the interaction is shown below:

**Table:** 

	*Lactic acid*	*Alcohol*
T1	1.412	1.413
T2	1.704	0.663
T3	1.316	1.232

#### Prevalence of AMR bacteria detected on hands

During the 2-week study, a total of 104 samples from both T1 and T3 were investigated for the presence of AMR bacteria (including MR-CoPS, AMR Enterobacteriaceae spp., and *Pseudomonas* spp.). For each participant, the samples were pooled for each product at each time point. MRSA was recovered from 14 (one nurse, four students, and nine veterinarians) and MRSP was cultured from five study participants (one receptionist, one student, and three veterinarians) (See overview Supplementary Table S1). Within T1 samples, the prevalence of AMR Enterobacteriaceae spp. was greatest (18%), followed by MR-CoPS (7%), with this pattern reversed at T3, when the MR-CoPS prevalence was greater compared with AMR Enterobacteriaceae spp. (13% compared to 11%, respectively) (Table 3). The differences in the prevalence of these resistant bacteria at T1 and T3 were not statistically significant (Supplementary Table S4).

**Table 3.** Number (N) and Prevalence (%), Including 95% Confidence Intervals of Participants with Hand Plates Positive for MR-CoPS, AMR *Enterobacteriaciae* spp., or *Pseudomonas* spp. Detected on Hands at T1 and T3 for Each Tested Product

	*Number*						
	*Prevalence (95% CI)*						
*Time period*	*T1*			*T3*			
*Hand hygiene product*	*Lactic acid(*n* = 52)*	*Alcohol(*n* = 52)*	*Total(*n* = 104)*	*Lactic acid(*n* = 52)*	*Alcohol(*n* = 52)*	*Total(*n* = 104)*	*Overall T1& T3(*n* = 208)*
MR-CoPS	4	3	7	5	8	13	20
	7% (3–18)	6% (2–16)	7% (3–13)	10% (2–10)	15% (8–28)	13% (7–20)	10% (6–14)
MRSA	3	2	5	4	5	9	14
	6% (2–15)	4% (1–13)	5% (2–10)	10% (4–20)	10% (4–20)	9% (4–15)	7% (4–11)
MRSP	0	1	1	2	2	4	5
		2% (0–10)	1% (0–5)	4% (1–13)	4% (1–13)	4% (2–9)	2% (1–6)
AMR Enterobacteriaceae spp.	9	10	19	2	9	11	30
	17% (9–30)	19% (10–32)	18% (12–27)	4% (1–13)	17% (9–30)	11% (6–18)	14% (10–20)
3GCR^1^	7	6	13	2	6	8	21
	13% (7–25)	12% (5–23)	13% (7–20)	4% (1–13)	12% (5–23)	8% (4–14)	10% (7–15)
MDR	2	1	3	0	3	3	6
	4% (1–13)	2% (0–10)	3% (1–8)		6% (2–16)	3% (1–8)	3% (1–6)
*bla*_TEM_^*1,2*^	3	1	4	0	1	1	5
	6% (2–16)	2% (0–10)	4% (2–9)		2% (0–10)	1% (0–5)	2% (1–6)
*bla*_SHV_^1,2^	2	0	2	0	1	1	3
	4% (1–13)		2% (1–7)		2% (0–10)	1% (0–5)	1% (0–4)
*bla*_CTX-M_^1^	3	0	3	0	0	0	3
	6% (2–16)		3% (1–8)				1% (0–4)
*qnrA^1^*	1	0	1	0	1	1	2
	2% (0–10)		1% (0–5)		2% (0–10)	1% (0–5)	1% (0–3)
*qnrS^1^*	3	1	2	0	6	6	8
	6% (2–16)	2% (0–10)	2% (1–7)		12% (5–23)	6% (3–12)	3% (2–7)
*Pseudomonas* spp.	1	2	3	0	2	2	5
	2% (0–10)	4% (1–13)	3% (1–8)		4% (1–13)	2% (1–7)	2% (1–6)

^1^ Phenotypic resistance to cefpodoxime (CPD).

^2^ These genes not sequenced to determine if they were ESBL gene variants.

N, number of positive samples; n, number of participants; CI, confidence interval; T1—after patient/environmental contact and before HH; T3—end of the day, before leaving the work place; AMR, antimicrobial resistant; MR-CoPS, methicillin-resistant coagulase-positive staphylococci; MRSA, methicillin-resistant *Staphylococcus Aureus*; MRSP, methicillin-resistant *Staphylococcus pseudintermedius*; AMR Enterobacteriaceae spp., Enterobacteriaceae spp. isolates resistant to at least one tested antimicrobial; 3GCR, third-generation cephalosporin resistance; MDR, multidrug resistant (resistant to three or more antimicrobial classes).

Enterobacteriaceae resistant to third-generation cephalosporins (3GCR) were recovered from the hands of 13 (13%) and 8 (8%) participants at T1 and T3, respectively. The overall prevalence of MDR Enterobacteriaceae spp. on participant's hands in this study was 3%. The genes encoding for antimicrobial resistance *bla*_TEM,_ *bla*_SHV_, *bla*_CTX-M_, *qnrA*, and *qnrS* were rarely detected. Carriage of *bla*_OXA_, *bla*_AmpC_, and *qnrB* was not detected in this study. Finally, five participants (three at T1 and two at T3) carried *Pseudomonas* spp. on their hands, giving an overall carriage prevalence of 2%. Full details can be found in Table 3.

## Discussion

This study provides the first evidence, to our knowledge, that an alcohol hand rub is superior at reducing bacterial counts on hands of healthcare professionals immediately after application compared to a lactic acid product. Previous work found that alcohol-based hand rubs are highly effective,^41^ they are microbiologically more effective than hand washing,^42^ and they are convenient and gentle to the skin,^43^ thus increasing compliance with HH protocols compared to hand washing.^44^ In addition, this is also the first time that the residual action of different HH rubs has been assessed. Although the lactic acid hand rub showed significant residual action 1 hour after application within a pilot study (Supplementary Table S2), neither product demonstrated residual antimicrobial effects by the end of a day of routine hospital practice. This highlights the importance of reapplying an HH rub after all procedures.

The discrepancy between the preliminary results and main study findings was unexpected. Lactic acid has been shown to be as effective as ethanol against *Escherichia coli in vitro* (unpublished data). A possible explanation for reduced immediate efficacy of the lactic acid HH rub is that the product had not completely dried on hands before they were imprinted onto the blood agar plate. Alcohol evaporates faster than water and thus, bacteria may be more easily transferred from hands when the skin is wet.^45^ Although participants were instructed to wait until hands were completely dry (60 seconds as per manufacturer's instructions) before imprinting their hands onto plates, they reported a rather “sticky” texture in their hands after the lactic acid HH rub was applied, suggesting that hands had not totally dried. As being “too busy” is perceived as an obstacle to good adherence to HH procedures by veterinary healthcare professionals,^27^ faster drying agents are likely to be more accepted and effective, as compliance has been suggested to be more important than the intrinsic activity of products themselves.^22^

The type of contact impacted contamination, with higher CFU after clinical staff handled patients compared to the environment. This contrasts to one study in human medicine where (nonsignificant) lower bacterial counts were identified after patient contact.^22^ This may be due to higher bacterial counts in animal compared to human patients, differing levels of contamination in hospital environments, or differences in definitions of environmental contact between studies.

Profession also impacted the degree of hand contamination, with clinicians having significantly higher CFU counts compared to nurses. Although some studies in human medicine^22,46^ found no correlation between job title and degree of hand contamination, other studies have shown nurses to be better at performing HH compared to clinicians,^47,48^ in accordance with our findings. The reasons for poor clinician compliance with HH compared to other professional categories remain unknown. However, previous studies^48^ found marked improvement in HH behavior following targeted clinician face to face educational campaigns; this will be taken into account in future educational programs in our institution.

In humans, studies evaluating differences in hand contamination between genders have yielded contrasting results, with some finding no disparity^22^ and others reporting the hands of female staff being significantly less contaminated compared to male staff.^46^ Gender was not associated with significant differences in CFU counts in our study. This was surprising, considering that the best group at performing HH was nurses and 10 of the 12 nurses enrolled in this study were female. However, a larger sample size may have produced different results. Further studies are needed to confirm this finding.

The MRSA and MRSP prevalences in this study were 7% and 2%, respectively. This is of particular concern, as colonization by MR-CoPS in veterinary clinical practice is reported to be a professional hazard.^49^ In fact, *S. pseudintermedius* is the leading opportunistic pathogen in dogs and MRSP, usually MDR, has rapidly spread in canine populations.^6^ To our best knowledge, the majority of studies in veterinary staff have investigated nasal carriage of MRSP, reporting carriage rates ranging from 0% to 3.9%.^49^ In a recent study, MRSP was found on the hands of 2 of 43 veterinary staff (5%) investigated.^50^ Two studies investigating the prevalence of MRSA hand carriage within the human medical profession reported similar results (5%)^51,52^ to our study.

In total, 30 participants (prevalence 14%) had their hands contaminated by AMR Enterobacteriaceae. Hand contamination with such bacteria is not surprising given that they are shed in faces of both community and hospitalized small animals.^53^ Enterobacteriaceae spp. are major causes of surgical site infections and catheter-associated urinary tract infections.^11^ Isolates resistant to third-generation cephalosporin as indicated by cefpodoxime resistance were recovered from the hands of 21 participants; in six of these people, the isolates were multidrug-resistant organisms and therefore resistant to most, if not to all, antibiotic classes used in small animal practice.^54^ Again, to the author's knowledge, there are no comparative studies in the literature.

This study has limitations. First of all, only potential pathogenic AMR bacteria, and not the total hand microbial flora, were characterized. However, our aim was not to characterize the total hand flora of veterinary staff and students, but only to get an idea of the prevalence of drug-resistant bacterial carriage. Another limitation due to time and financial constraints is that not all available isolates were further characterized. Due to the initial hypothesis that the HH rubs would be effective at reducing microbial flora immediately after application, thus expecting fewer counts at T2, the study protocol was designed so that further characterization of the isolates was performed only at T1 (before HH) and T3 (6–8 hours after first HH). Retrospectively, and due to the lack of expected efficacy of one of the products, it would have been interesting to be able to characterize the isolates obtained immediately after HH (T2). Finally, it is important to bear in mind that the findings in this veterinary referral teaching hospital may not be fully representative for first opinion, nonteaching settings.

In conclusion, the tested alcohol-based hand rub was effective at reducing bacterial counts on veterinary hospital staff hands, but did not have residual action, highlighting the importance of repeated application. In contrast, the lactic acid rub was less effective than anticipated. AMR bacteria were found on hands of staff before using HH products; in particular, being a veterinarian rather than a nurse and touching a patient rather than the environment were significantly associated with increased hand contamination. Further studies evaluating the potential residual action of hand rub products, and ways to improve HH compliance among veterinary professionals, in particular clinicians, are needed.

## Supplementary Material

Supplemental data
