# Supplementary material for: Are the Hands of Veterinary Staff a Reservoir for Antimicrobial-Resistant Bacteria? A Randomized Study to Evaluate Two Hand Hygiene Rubs in a Veterinary Hospital
Source: Microb Drug Resist. 2018 Dec 8;24(10):1607–16. doi: 10.1089/mdr.2018.0183 (PMC6306656; doi:10.1089/mdr.2018.0183)
Supplement: Supplemental data [file Supp_Data.pdf]

Supplementary Data

Preliminary Study 1

A preliminary study was used to assess the potential residual action of the lactic acid hand rub (LA-HH) rub under controlled circumstances. Briefly, 20 volunteers were asked to vigorously rub their own face/neck and to perform hand hygiene (HH) with the LA-HH rub immediately after. The volunteers were sampled on three occasions: immediately after rubbing their face/neck (T1), 60 seconds after performing the HH procedure (T2), and 1 hour later (T3). The whole process was repeated on three consecutive days, in the presence of one of the investigators. Participants were asked not to wash their hands between T2 and T3 so that the residual action of the product could be evaluated. The participant sampling, the processing of plates, and analysis were performed as described in the Methods section.

The LA-HH achieved a significant reduction in median colony-forming units (CFU) per hand 1 hour after application ($p < 0.05$). In contrast, the median CFU per hand 60 seconds after application of the product was not significantly different compared to before the HH procedure ($p = 0.9$) (Supplementary Table S2).

Preliminary Study 2

The second preliminary study investigated the potential reduction in bacterial CFU after the use of both the alcohol hand rub (A-HH) and the LA-HH; these results were then utilized within sample size calculations. Ten volunteers were asked to rub their own face/neck and then perform HH with the two HH rubs, on two separate occasions. Following HH, the volunteers imprinted their dominant hand (finger tips and thumb) into Columbia 5% defibrinated horse blood agar (CAB) plates supplemented with neutralizer (30 mL/L Tween 80, 30 g/L Saponin, 1 g/L L-histidine, and 1 g/L L-cysteine). The process was observed by a study investigator, and plates were processed and read as described in the Methods section. The mean CFU after the A-HH rub was 86.7 and after the LA-HH was 206.80.

Supplementary Table S1. Overview of Colony-Forming Units Per Hand of Each Participant and of the Presence/Absence of Antimicrobial-Resistant Bacteria Before Performing Hand Hygiene (T1) and at the End of the Day, Before Going Home (T3), Stratified by Profession and Hand Hygiene Rub Usage

		CFU/hand[a] *A-HH*			*CFU/hand*[a] *LA-HH*			*T1 A-HH*				*T1 LA-HH*				*T3 A-HH*				*T3 LA-HH*			
	Participants	*T1*	*T2*	*T3*	*T1*	*T2*	*T3*	*MRSA*	*MRSP*	*AMR Enterobacteriaceae*	*Pseudomonas spp.*	*MRSA*	*MRSP*	*AMR Enterpbacteriaceae*	*Pseudomonas spp.*	*MRSA*	*MRSP*	*AMR Enterobacteriaceae*	*Pseudomonas spp.*	*MRSA*	*MRSP*	*AMR Enterobacteriaceae*	*Pseudomonas spp.*
Reception	1	60	14	132	245	350	298							+				+					
	2	126	66	59	159	257	136			+													
	3	103	69	30	15	129	94																
	4	36	15	28	58	144	28														+		
Auxiliaries	5	268	14	32	69	276	61																
	6	248	152	253	214	350	266																
	7	64	93	235	253	332	253																
	8				350	67	62																
Nurses	9	27	12	29	39	313	52															+	
	10	89	2	22	189	235	157			+								+	+				
	11	26	6	11	27	57	1			+	+			+									
	12	279	73	24				+															
	13	74	6	198	103	109	56																
	14	48	57	2	117	69	9																
	15	16	5	4	69	38	62																
	16	9	0	5	37	16	17																
	17	45	0.5	30	157	288	96			+													
	18	88	48	16	5	64	17																
	19	28	15	156	26	19	26																
	20	20	2	22	63	106	22							+									
Students	21	123	143	106	42	266	188																
	22	84	5	90	165	208	85							+				+					
	23	59	26	50	39	25	24											+					
	24	47	15	96	84	191	83								+								
	25	202	190	161	115	177	80			+						+							
	26	110	21	103	79	210	27										+						
	27	81	19	166	18	83	18																
	28	101	36	96	28	236	263																
	29	192	20	203	152	332	54																
	30	97	3	5	269	106	161			+													
	31	106	4	98	308	284	39							+									
	32	150	30	77	231	350	281			+													
	33	123	54	289	61	71	333							+		+							
	34	224	8	102	178	208	82					+								+			
Veterinarians	35	103	3	106	74	189	85																
	36	268	18	210	196	270	181																
	37	51	20	30	240	91	11							+									
	38	72	131	42	95	262	108					+						+					
	39	188	120	143	214	235	270											+					
	40	261	26	279	209	302	81									+							
	41	29	3	134	64	350	142	+				+				+				+			
	42	137	20	62	39	295	39																
	43	290	74	350	231	350	152							+						+		+	
	44	18	8	6	25	169	13													+			
	45	3	0	30	21	259	12		+								+						
	46	9	0	5	61	152	99														+		
	47	191	27	214	155	234	166			+								+					
	48	7	1	8	38	11	18																
	49	275	59	84	305	350	310			+				+									
	50	128	19	34	31	137	29																
	51	62	27	231	300	233	276											+	+				
	52	88	8	1	33	85	77			+	+												
	53	321	131	350	95	123	138											+					

CFU, colony-forming units; A-HH, Alcohol hand rub; LA-HH, lactic acid hand rub; T1—after patient/environmental contact and before HH, T2—immediately after HH, T3—6–8 hours later, just before going home; MRSA, methicillin-resistant *Staphylococcus Aureus*; MRSP, methicillin-resistant *Staphylococcus pseudintermedius*; AMR *Enterobacteriaceae*: *Enterobacteriaceae* spp. isolates resistant to at least one tested antimicrobial

[a]Dominant hand.

Supplementary Table S2. Evaluation of the Potential Residual Action of Lactic Acid Using Examination of Median CFU Per Hand Obtained at 60 Seconds and 1 Hour After Hand Hygiene Procedure

	N *sample*	*Median CFU per hand*[a] *(interquartile range)*	p *value*[b]
T1	20	173 (186.91)	
T2	20	154 (229.42)	0.9
T3	20	49 (125.83)	**<0.01**

N, number of participants; T1—after vigorously rubbing own face/neck and before HH; T2—immediately after performing HH with lactic acid hand rub; T3—1 hour after T2.

[a]Dominant hand.

[b]p value for Wilcoxon signed rank test comparing CFU at time T2 and T3 compared to T1; significant if $p < 0.05$.

Supplementary Table S3. Multilevel, Univariable Regression Model Describing Factors Associated with Overall Hand Contamination After Hands Had Been Treated Using Either Lactic Acid- or Alcohol-Based Hand Hygiene Products

Term	*Beta*	*SE*	*Wald* p *value*
Gender			0.67
Female	Ref	–	
Male	–0.05	0.11	
Product			
Alcohol (ref)			
Lactic acid	0.40	0.04	<0.001
Day during the week			0.9
Day 1	Ref		
Day 2	–0.01	0.05	
Day 3	–0.01	0.05	
Type of contact			<0.001
Environment	Ref		
Animal	0.27	0.06	
Which product first			0.61
Alcohol			
Lactic acid	–0.06	0.11	
Job			0.014
Nurse	Ref		
Administration staff	0.31	0.20	
Auxiliaries	0.42	0.22	
Veterinarians	0.43	0.13	
Students	0.20	0.14	
Time			<0.001
T1	Ref		
T2	–0.23	0.05	
T3	–0.14	0.05	
Surgeon			0.56
No	Ref		
Yes	0.11	0.19	

The outcome is the log colony-forming units, and the model includes clustering within participants.

Ref, used as reference in the multilevel modeling; SE, standard error; significant if $p < 0.05$. T1—after patient/environmental contact and before HH, T2—immediately after HH, T3—6–8 hours later, just before going home.

Supplementary Table S4. Comparison of the Prevalence of MR-CoPS and AMR *Enterobacteriaceae* spp.

	N		
	Prevalence		
	T1 (n = *104)*	*T3* (n = *104)*	p *value*
MR-CoPS	7<br>7%	13<br>13%	0.109
AMR *Enterobacteriaceae* spp.	19<br>18%	11<br>11%	0.152

On study participants' hands between T1 and T3.

N, number of positive samples; n, total number of participants (52 pooled samples from participants from the lactic acid group and 52 pooled samples from participants from the alcohol group); T1—after patient/environmental contact and before HH, T2—immediately after HH, T3—6–8 hours later, just before going home; MR-CoPS, methicillin-resistant coagulase-positive staphylococci; AMR *Enterobacteriaceae* spp., *Enterobacteriaceae* spp. isolates resistant to at least one tested antimicrobial; *p* value for McNemar's test comparing prevalence of MR-CoPS and AMR *Enterobacteriaceae* spp. at T1 compared to T3; significant if $p < 0.05$.
